# Integrative analysis of RNA-seq and Ribo-seq reveals that lncRNA regulates chicken myogenesis through encoding peptide

**DOI:** 10.1186/s40104-026-01421-y

**Published:** 2026-05-29

**Authors:** Yufang Niu, Liyang He, Zhiyuan An, Meng Yuan, Haigang Ji, Wei Wang, Chengjie Wei, Ruili Han, Weihua Tian, Yadong Tian, Xiaojun Liu, Xiangtao Kang, Zhuanjian Li

**Affiliations:** 1 College of Animal Science and Technology, Henan Agricultural University, Zhengzhou, 450046 China; 2 Key Laboratory of Livestock and Poultry Resources (Poultry) Evaluation and Utilization of Ministry of Agriculture and Rural Affairs, Henan Agricultural University, Zhengzhou, 450046 China

**Keywords:** Differentiation, LncRNA, Myoblasts, MPD-74aa, Proliferation

## Abstract

**Background:**

Long noncoding RNAs (lncRNAs) participate in various critical regulatory steps during myogenesis. LncRNAs can encode small peptides, which can regulate gene expression through multiple mechanisms, thereby participating in key biological processes.

**Results:**

The lncRNAs were screened between proliferating and differentiating myoblast in chicken through RNA-seq and Ribo-seq. As a result, 178 DE-lncRNAs were identified in RNA-seq, and three of them were identified as differentially translated lncRNAs by Ribo-seq. Among them, lncMPD, which showed coding potential, was highly expressed in proliferating myoblast. It encoded a small peptide containing 74 amino acids, which was named MPD-74aa. MPD-74aa was validated via WB and mass spectrometry. We subsequently confirmed that MPD-74aa promotes myoblast proliferation and inhibits its differentiation. Co-IP revealed that MPD-74aa interacts with the protein CDK1. Moreover, MPD-74aa positively regulated the expression of CDK1.

**Conclusion:**

This study confirms that the lncMPD plays a crucial regulatory role in the chicken myogenesis by encoding the small peptide MPD-74aa. Mechanistically, MPD-74aa exerts its regulatory function through interaction with CDK1, a key protein marker of cell proliferation. These findings provide new insight into the molecular mechanisms about the coding capacity of lncRNA regulating chicken muscle development.

**Supplementary Information:**

The online version contains supplementary material available at 10.1186/s40104-026-01421-y.

## Introduction

Skeletal muscle is the largest tissue in the animal body, accounting for approximately 40% of the total body mass, and is a major participant in regulating energy homeostasis [1–3]. Meanwhile, skeletal muscle is also the largest metabolic and endocrine organ in the animal body, playing a crucial role in animal movement, protein storage, metabolism, and visceral protection [4]. Skeletal muscle development is a complex process that primarily involves the proliferation, differentiation, and fusion of myoblasts to form multinucleated myotubes that eventually develop into mature muscle fibers. As the pivotal precursor cells for muscle development, myoblasts represent an ideal in vitro model for investigating myogenesis. The process of skeletal muscle development is precisely regulated by a series of regulatory factors and signaling pathways [5–7], among which the myogenic regulatory factor family (including *MYOD* [8], *MYF5* [9], *MYOG* [10] and their coregulator MEF2 [11]) plays a central role during differentiation.

Studies have shown that the interaction between *MYOD* and the JNK/MAPK signaling pathway affects myoblast differentiation [12, 13]. MAPK family members such as ERK [14] and p38 MAPK [15, 16] also participate in the regulation of myogenic transcription factor activity, muscle-specific gene expression, and myofiber formation at different stages. Through synergistic or antagonistic crosstalk with the Notch, Wnt/β-catenin, and TGF-β/Smad pathways, they collectively control the proliferation and differentiation of myogenic cells [17].

Cyclin-dependent kinase 1 (CDK1), an important cell cycle regulator, plays a key role in driving cell cycle progression and maintaining cell proliferation [18]. Current studies show that CDK1 participates in skeletal muscle development by regulating skeletal muscle satellite cell proliferation, muscle fiber hypertrophy and post-injury regeneration [19].

LncRNAs are a new type of noncoding RNAs with a length of more than 200 nucleotides. They have been identified, and their functions have been verified in various cells; moreover, lncRNAs play important biological roles in skeletal muscle development and disease processes [20–23]. Numerous studies have shown that lncRNAs are deeply involved in the regulation of myoblast proliferation and differentiation through diverse molecular mechanisms [24, 25]. Additionally, lncRNAs can modulate other physiological and pathological processes through distinct pathways; for instance, they participate in myocardial fibrosis, regulation of uterine myometrial contraction, and cancer metabolic remodeling in an m^6^A-dependent manner [26–30].

The continuous advancement of high-throughput sequencing technologies has facilitated the combined application of RNA-seq and Ribo-seq, which has emerged as a core tool for deciphering gene expression profiles and translational dynamics. Notably, this technological combination has accelerated the discovery of noncanonical small open reading frames (sORFs), further verifying that certain lncRNAs can encode small peptides [31–35]. Translated from sORFs, these lncRNA-encoded small peptides [36–39], have specific and critical biological functions: they can act as oncogenic drivers or tumor suppressors to regulate multiple cancer-related processes [40–48], are immunogenic enough to elicit robust antitumor responses [49], and even modulate autoimmune responses [50]. However, the functional lncRNA-encoded small peptides that govern skeletal muscle growth and development remain incompletely understood. Thus, identifying and conducting in-depth investigations into lncRNA-encoded small peptides that exert key regulatory effects on myoblast proliferation and differentiation is highly important for refining the epigenetic regulatory network underlying skeletal muscle development.

Therefore, via combined analysis of RNA-seq and Ribo-seq data, this study identified and thoroughly investigated lncRNAs and their encoded peptides that play crucial regulatory roles in myoblast proliferation and differentiation, aiming to elucidate the molecular regulatory network involved in chicken skeletal muscle development, providing novel targets and theoretical support for molecular breeding in poultry.

## Materials and methods

### Cell culture

Myoblasts were isolated from the leg muscles of 11-day-old Arbor Acres (AA) broilers. The detailed program was described by Niu et al. [51]. The isolated cells were maintained in basal culture medium consisting of Dulbecco’s Modified Eagle Medium (DMEM) (Gibco, USA). The concentration of fetal bovine serum (Gibco, USA) in this medium was 15%, and the concentration of penicillin‒streptomycin (Gibco, USA) was 1%. The cells were cultured in a humidified cell incubator at 37 °C with 5% CO_2_. When the cell density reached 100%, the myoblasts were placed in differentiation medium (high-glucose DMEM supplemented with 2% horse serum (Gibco, USA) and 1% penicillin/streptomycin). The myoblasts were collected at 50% and 100% confluence, as well as at days 1, 2, 4, 6, and 8 post-differentiation (D1, D2, D4, D6, and D8), with three biological replicates per group for whole-transcriptome sequencing.

### RNA-seq and Ribo-seq

RNA-seq was performed on myoblasts from seven developmental stages, with the detailed sequencing and analytical protocols conducted as described by Niu et al. [51].

Ribo-seq was performed as follows: Cells harvested at specific time points were treated with trypsin for translational inhibition and fixation and then digested with RNase to remove unprotected RNA fragments, thereby isolating ribosome-protected fragments. Ribosome-protected 30 bp RNA fragments were sequenced using an Illumina NovaSeq X Plus. Clean reads were aligned to the chicken ribosome using Bowtie2 (v2.2.8); ribosomal RNA-mapped reads were excluded, and 20‒40 bp reads were retained. DESeq2 identified significantly differentially expressed genes (FDR < 0.05, |log_2_fold change| > 1). Potential sORFs in noncoding regions of reference genome–derived transcripts were searched, and uniquely mapped RF reads (Bowtie2) were used to calculate ORF FPKM values. EdgeR was used to analyze the differential translation of sORFs (|log_2_fold change| ≥ 1, FDR < 0.05), and the correlation between the fold changes in uORF and mORF translation was evaluated. The sORFs with average FPKMs ≥ 1 were annotated for translational potential (Fickett_score > 0.74, RRS > 1.0245, ORF score > 3.74). Functional domains were predicted by aligning protein sequences against pfam (v26.0).

### Construction of overexpression vectors and cell transfection

The predicted MPD-74aa sequence was amplified from the cDNA of leg muscles by PCR. The full-length amplified sequence of MPD-74aa was inserted into the pcDNA3.1–3 × Flag vector. Myoblast transfection was performed using Lipofectamine 3000 (Thermo Fisher Scientific, USA).

### RNA extraction, cDNA synthesis and quantitative real-time PCR (qRT-PCR)

Tissue and myoblast samples were homogenized in TRIzol reagent (Vazyme Biotech, China) for total RNA isolation. Purity and integrity were assessed using a NanoDrop 2000 spectrophotometer (Thermo Fisher Scientific, USA). RNA was reverse transcribed into cDNA with a HiScript kit (Vazyme Biotech, China) following the supplier’s protocol. Amplifications were carried out in triplicate using SYBR Green chemistry on a LightCycler 96 platform (Roche Applied Science, Switzerland). *GAPDH* served as an endogenous control for mRNA expression. Relative expression levels were calculated using the 2^−ΔΔCt^ method; the primer sequences are shown in Table S1.

### Western blot (WB) analysis

Cells were then washed three times with Tris-buffered saline (Beyotime, China). Total cellular proteins were extracted using RIPA buffer (Beyotime, China) supplemented with protease and phosphatase inhibitor reagents (Thermo Fisher Scientific, USA). The protein concentration was determined using a BCA protein quantification assay kit (Beyotime, China). The total proteins were boiled for 10 min for denaturation and then separated on a 10% sodium dodecyl sulfate–polyacrylamide gel electrophoresis (SDS-PAGE) gel. The separated proteins were subsequently transferred to a methanol-activated polyvinylidene fluoride membrane (Millipore, USA). Next, the membrane was blocked with 5% nonfat milk powder containing 0.05% Tween-20 for 1 h and then incubated with primary antibody overnight at 4 °C. After that, the membrane was washed three times with Tris-buffered saline containing Tween-20 for 5 min each and then incubated with the secondary antibody for 1 h at room temperature. An ECL Plus kit (Beyotime, China) was used to identify the protein bands. The gray values for each band were calculated using ImageJ software (NIH, USA). The protein expression was normalized to that of β-actin as a loading control. MYHC antibody (1:400, B103) was purchased from Xiandu Biotechnology (DSHB, USA). β-Actin (1:5,000, 66009-1-Ig), CDK1 (1:2,000, 19532-1-AP), and DYKDDDDK (1:5,000, 66008-4-Ig) antibodies were purchased from Proteintech (Wuhan, China).

### Cell Counting Kit-8 (CCK-8)

Cells were seeded and cultured in a 96-well plate. When the cell density reached 70%‒80%, the target vector and the empty vector were transfected into the cells using Lipofectamine 3000 (Thermo Fisher Scientific, USA). At 12, 24, 36, and 48 h after transfection, 10 μL of CCK-8 reagent (Vazyme Biotech, China) was added to each well, followed by incubation in the dark for 2 h. The absorbance at 450 nm was measured using a microplate reader (Thermo Fisher Scientific, USA), and the cell viability was calculated.

### Flow Cytometry-based cell sorting

Cells were seeded and cultured in a 6-well plate. When the cell density reached 70%‒80%, the target vector and empty vector were transfected into the cells using Lipofectamine 3000 (Thermo Fisher Scientific, USA), after which the cells were placed back into the incubator for 24 h. The adherent cells were digested with 0.25% EDTA digestive solution (Solarbio, China), washed twice with PBS (Solarbio, China), fixed with 70% alcohol, and stored at 4 °C (Gree Electric Appliances, China) overnight. The samples were sent to Wuhan Servicebio Technology for cell cycle detection.

### Cell proliferation assays

The 5-Ethynyl-2-deoxyuridine (EdU) assay was performed according to the instructions of the EdU kit (RiboBio, China). Images were captured using a fluorescence microscope (Olympus, Japan), and the proliferation rate was quantified with ImageJ software (*n* = 3).

### Immunofluorescence

Cells were seeded in 12-well plates, and immunofluorescence analysis of MYHC was performed with anti-MYHC antibody and anti-mouse Cy3-conjugated antibody (1:80; Proteintech, China). Nuclear staining was performed with DAPI (Solarbio, China). The images were obtained under a fluorescence microscope (Olympus, Japan) (*n* = 3).

### Coimmunoprecipitation (Co-IP) and mass spectrometry

For immunoprecipitation, 50 µL of Pierce DYKDDDDK Magnetic Agarose slurry (Thermo Fisher Scientific, USA) was mixed with 450 µL of binding buffer (Thermo Fisher Scientific, USA), and the tube was placed on a magnetic stand to collect the beads. After the supernatant was discarded, the beads were washed twice with 500 µL of binding buffer. Prewashed agarose was then mixed with pcDNA3.1-3×Flag-MPD-74aa (OV-MPD-74aa) samples by gentle vortexing or inversion, followed by incubation at room temperature for 20 min with continuous mixing to allow MPD-74aa-interacting proteins to bind. The beads were collected via magnetic stand, sequentially washed with 500 µL of PBS and 500 µL of purified water, and then eluted with 100 µL of elution buffer (pH 2.8; Thermo Fisher Scientific, USA) for 5 min at room temperature. The eluted supernatant was collected, neutralized with 15 µL of neutralization buffer per 100 µL of extract, and boiled in SDS-PAGE sample buffer for 10 min. Proteins were separated by 10% SDS-PAGE and visualized using mass spectrometry-compatible silver staining (Invitrogen, USA). LC-MS/MS data were acquired with an Orbitrap Exploris 480 mass spectrometer (Thermo Fisher Scientific, USA) and analyzed by Mascot server software (Matrix Science, UK), with peptide search criteria of confidence ≥ 95% and unique peptides ≥ 1.

Total cellular protein was extracted from untreated myoblasts. After Coomassie brilliant blue staining, proteins smaller than 20 kDa were subjected to mass spectrometry. Raw mass spectrometry data were analyzed using MaxQuant software (v1.5.2.8) [52], and peptide sequences were aligned against the chicken UniProt database.

### Statistical Analysis

All the data are presented as the mean ± SEM. Statistical analyses of the data were performed using GraphPad Prism 7.0 software (GraphPad Software, USA). Differences between the two groups were analyzed using *t*-tests (^*^P < 0.05, ^**^P < 0.01, ^***^P < 0.001, ns means not significant).

## Results

### Screening of skeletal muscle development-related lncRNAs by RNA-seq

**Fig. 1:**
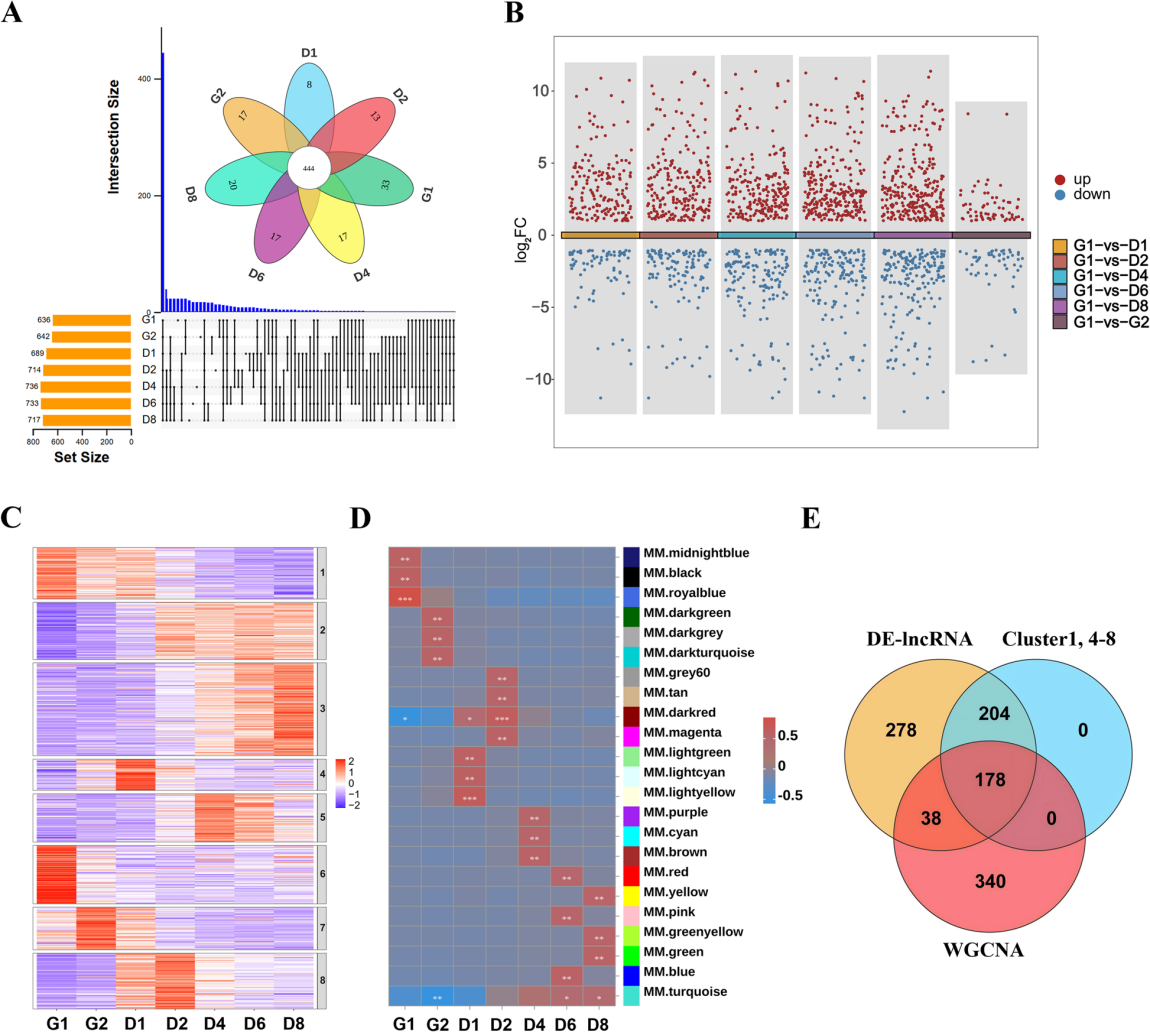
Screening of skeletal muscle development-related lncRNAs by RNA-seq. **A** Venn diagram of lncRNAs at different stages (G1: Myoblasts were grown to 50% confluence; G2: Myoblasts were grown to 100% confluence; D1, D2, D4, D6, D8: 1, 2, 4, 6, 8 d after post-differentiation). **B** DE-lncRNAs multigroup scatter plot. **C** K-means clustering analysis of DE-lncRNAs. **D** Heatmap of module correlations across different developmental stages. **E** Venn diagram of the results of the DE-lncRNAs analysis, k-means clustering analysis, and WGCNA

In this study, we collected samples at seven different stages during myoblast development and conducted RNA sequencing analysis. Through cross-stage comparisons, a total of 990 lncRNAs were identified, among which 444 were commonly expressed in all seven stages (Fig. 1A and Table S2). Differential analysis using the G1 phase as a reference yielded 1,984 differentially expressed lncRNAs (DE-lncRNAs), and after removing redundant entries, 698 unique DE-lncRNAs were retained (Fig. 1B). Expression distribution profiles were generated on the basis of the FPKM values to analyze expression patterns, revealing that lncRNAs in the G1 phase exhibited relatively high overall expression levels (Fig. S1A). GO and KEGG enrichment analyses of the target genes of DE-lncRNAs revealed that they were enriched mainly in pathways related to the cell cycle and DNA replication, indicating that these lncRNAs may regulate the proliferation of myoblasts (Fig. S1B and C). We further screened the DE-lncRNAs using k-means clustering analysis and identified lncRNA clusters (Clusters 1, 4, 5, 6, 7, and 8) that were highly expressed specifically during myoblast proliferation and differentiation (Fig. 1C). Weighted gene coexpression network analysis (WGCNA) was subsequently performed on all the identified lncRNAs to select the modules most strongly associated with myoblast proliferation and differentiation (Fig. 1D and Fig. S1D). Finally, the intersection of the DE-lncRNAs analysis, k-means clustering analysis, and WGCNA results was successfully used to screen and identify 178 target lncRNAs (Fig. 1E and Table S3).

### Screening coding-potential lncRNAs by RNA-seq and Ribo-seq integration

**Fig. 2:**
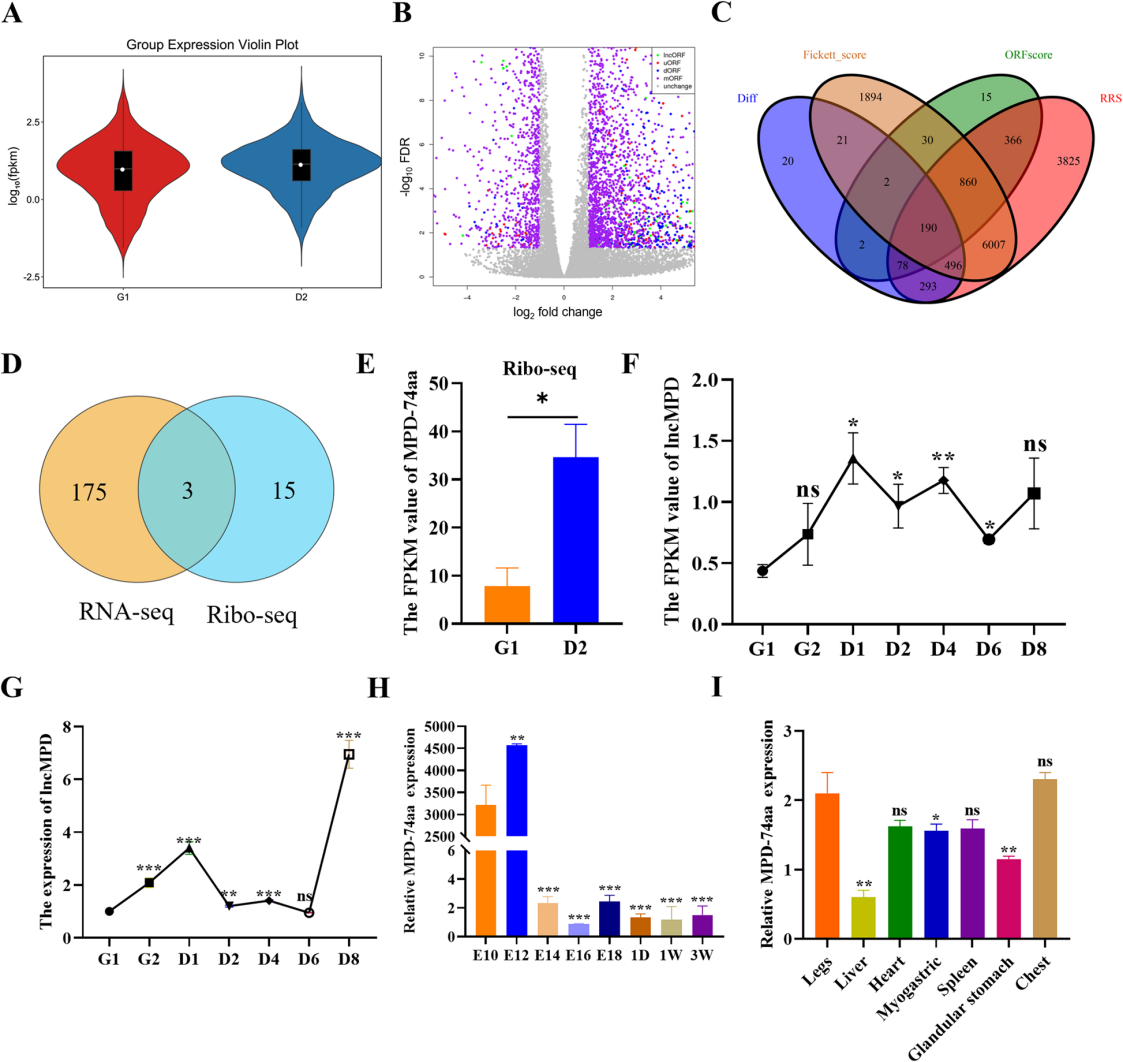
Screening coding-potential lncRNAs by RNA-seq and Ribo-seq integration. **A** Violin plots of sORF expression levels across groups. **B** Comparison of G1 vs. D2 differences in a volcano plot. **C** RRS results, ORF scores, Fickett_score, and differentially expressed sORFs combined analysis Venn diagram. **D** Venn analysis of DE-lncRNAs between Ribo-seq and RNA-seq. **E** Ribo-seq verification of MPD-74aa. **F** RNA-seq verification of lncMPD. All comparison groups were analyzed for differences relative to the G1 phase as the control (the same as below). **G** qRT-PCR verification of lncMPD. **H** Expression levels of MPD-74aa at different time points. All comparison groups were analyzed for differences relative to the E10 as the control. **I** Tissue expression profile of MPD-74aa at E12. All comparison groups were analyzed for differences relative to the legs as the control. Data are shown as the mean ± SEM (*n* = 3) (^*^P < 0.05, ^**^P < 0.01, ^***^P < 0.001, ns means not significant)

To investigate the coding potential of target lncRNAs, we performed Ribo-seq analysis on myoblasts in the G1 and D2 phases. Pearson correlation coefficient analysis of the Ribo-seq sample heatmap revealed high correlations within each sample group (Fig. S2A). Statistical analysis of the gene expression abundance distribution across all the samples revealed that overall gene expression levels were higher in the D2 phase than in the G1 phase (Fig. 2A). A total of 3,545 differentially translated genes were identified from the translatome data. Among them, 2,263 genes were upregulated, and 1,282 genes were downregulated. Further analysis revealed that these differentially expressed genes included several key marker genes closely related to cell proliferation and differentiation, such as *CDK1*, *PCNA*, *MYOD1*, and *MYOG* (Fig. S2B and Table S4). We subsequently analyzed the coding potential of the noncoding regions within the lncRNA transcripts and identified 9,299,922 sORFs. These included 86,419 lncORFs originating from lncRNA regions, 8,761 uORFs derived from gene 5' UTR, and 60,760 dORFs originating from gene 3' UTR regions. Differential analysis of these sORFs was subsequently performed, identifying 1,102 differentially expressed sORFs (Fig. 2B). On the basis of the Ribo-seq data, combined with the results of the ribosome release score (RRS) analysis and ORF scoring, 1,494 sORFs were selected (Fig. S2C). Further combining Fickett_scores and differentially expressed sORFs, 190 target sORFs were identified (Fig. 2C). Among these 190 sORFs, 47 ORFs originated from 18 lncRNAs.

To identify DE-lncRNAs with translational capacity between the G1 and D2 groups, we performed a joint analysis of 178 DE-lncRNAs identified by RNA-seq and 18 lncRNAs with coding potential screened by Ribo-seq. Consequently, 3 DE-lncRNAs (ENSGALT00000100638, ENSGALT00000095226, and ENSGALT00000097778) exhibited coding potential. Among these three candidates, ENSGALT00000100638, which was named as lncMPD (Myoblasts proliferation and differentiation), was highly expressed in proliferating myoblasts (Fig. 2D). We named its small peptide as MPD-74aa since it encoded 74 amino acids. Ribo-seq data revealed that MPD-74aa expression was significantly higher in the D2 phase than in the G1 phase (Fig. 2E), and the expression of lncMPD was significantly greater in the D2 phase than in the G1 phase (Fig. 2F). In addition, expression profile analysis revealed that lncMPD expression was significantly upregulated in the G2 phase (Fig. 2G); during embryonic development, MPD-74aa maintained relatively high expression abundance at the key time points E10 and E12 (Fig. 2 H). Furthermore, MPD-74aa was highly expressed in both the leg and pectoralis muscle tissues of E12 embryos (Fig. 2I). This expression pattern suggests that MPD-74aa may play important roles in the regulation of cell proliferation and the development process of skeletal muscle.

### lncMPD encodes a small peptide

**Fig. 3:**
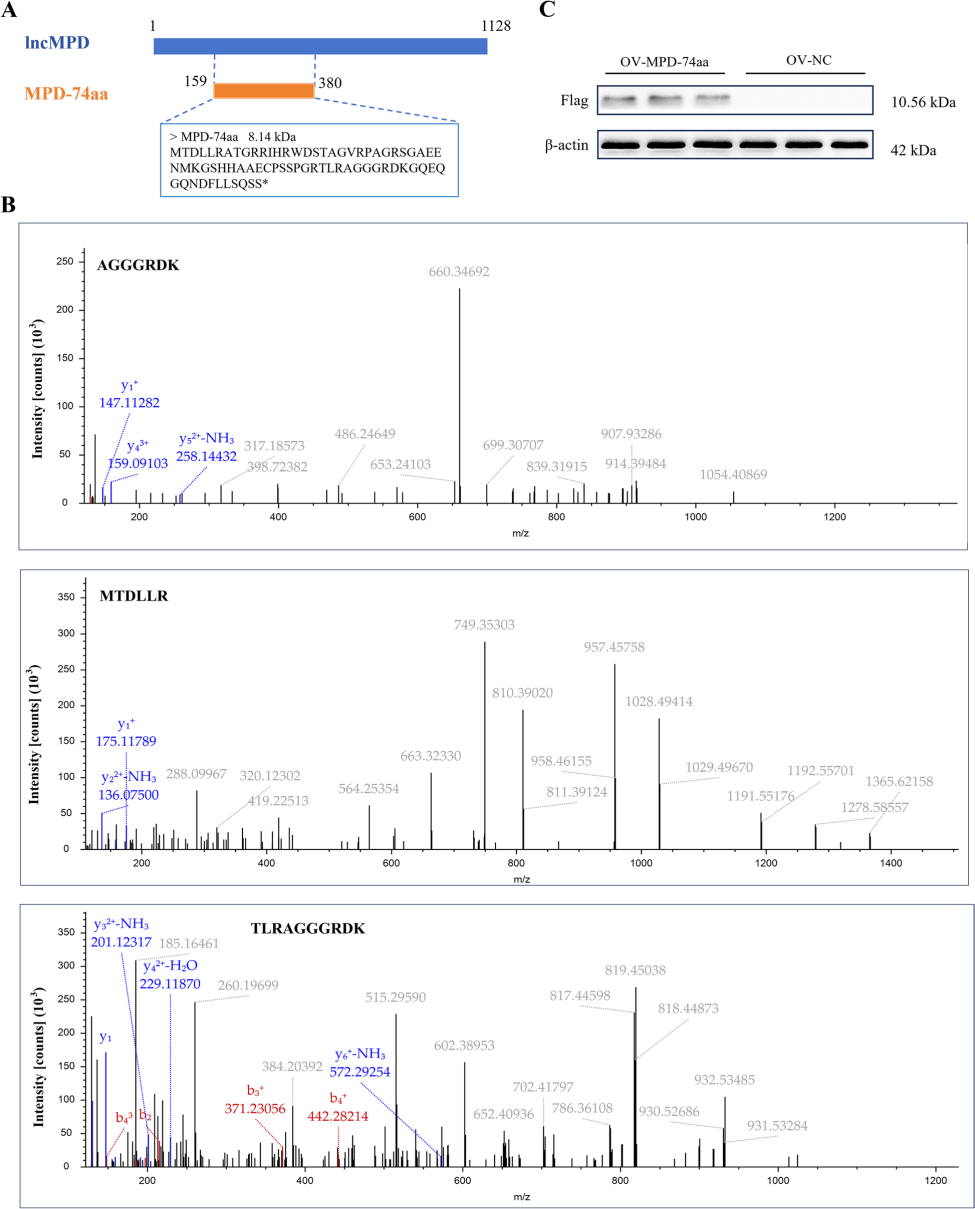
lncMPD encodes a small peptide. **A** Location information for MPD-74aa and the protein sequence it encodes. **B** Secondary mass spectrum of MPD-74aa. **C** Flag protein with a molecular weight of approximately 10.56 kDa was detected in the ov-MPD-74aa-transfected group by WB

To investigate whether MPD-74aa has coding potential, mass spectrometry analysis was performed in this study, and the results revealed significant enrichment of peptides corresponding to MPD-74aa (Fig. 3A and B). To further validate these findings, we performed Western blot analysis. On the basis of the positions of the protein marker bands, the target band appeared between 10 and 15 kDa, and after the insertion of the 3 × Flag, the size of the MPD-74aa protein was calculated to be 10.56 kDa according to the amino acid sequence (Fig. 3C).

### MPD-74aa promotes myoblast proliferation and inhibits differentiation

**Fig. 4:**
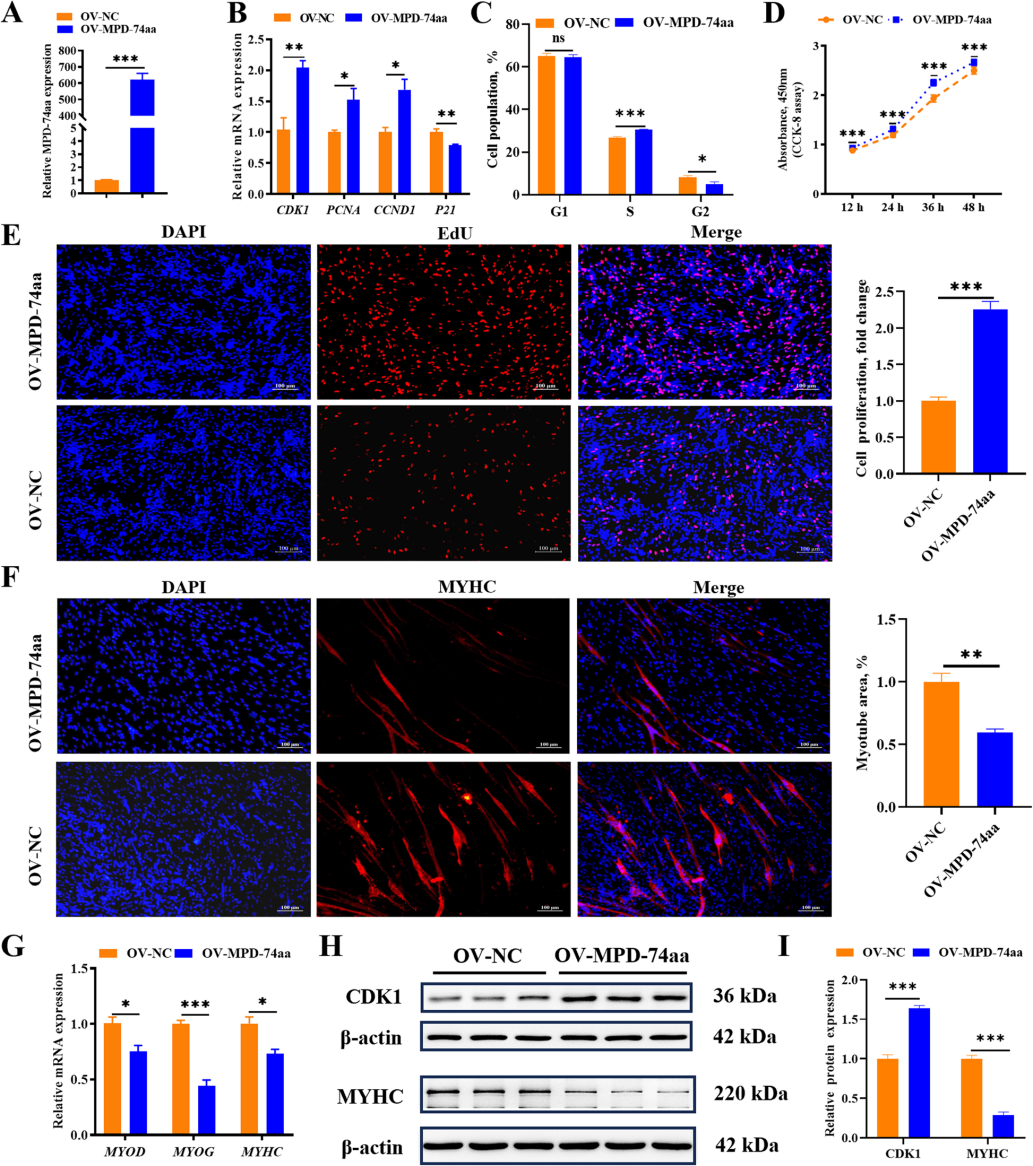
MPD-74aa promotes myoblast proliferation and inhibits differentiation. **A** Overexpression efficiency of MPD-74aa in myoblasts. **B** Effect of MPD-74aa overexpression on the mRNA expression of *CDK1*, *PCNA*, *CCND1*, and *P21*. The relative gene expression levels are shown as fold changes versus those in the control group. **C** Cell cycle analysis of chicken myoblasts upon MPD-74aa overexpression. **D** CCK-8 assay of proliferating myoblasts overexpressing MPD-74aa. **E** EdU staining analysis of proliferating myoblasts after MPD-74aa overexpression (scale = 100 μm). The percentages of EdU-positive cells are shown as fold changes versus those in the control group. **F** MYHC immunostaining of MPD-74aa-overexpressing chicken myoblasts at 48 h post-differentiation (scale = 100 μm). The proportion of immunostaining-positive cells is shown as the fold change versus that in the control group. **G** The mRNA expression levels of genes associated with myoblast differentiation, including *MYOD*, *MYOG*, and *MYHC*, in differentiated MPD-74aa-overexpressing myoblasts. **H** and **I** The protein expression of MYHC and CDK1 in differentiating and proliferating MPD-74aa-overexpressing chicken myoblasts. The relative protein expression levels are shown as fold changes versus those in the control group. Data are shown as the mean ± SEM (*n* = 3) (^*^P < 0.05, ^**^P < 0.01, ^***^P < 0.001, ns means not significant)

To investigate the biological function of MPD-74aa, we successfully transfected MPD-74aa-overexpressing vectors into myoblasts (Fig. 4A). Compared with control treatment, overexpression of MPD-74aa significantly increased the expression of proliferation-promoting genes (*CDK1*, *PCNA*, and *CCND1*) and decreased the expression of *P21* in chicken myoblasts (Fig. 4B). MPD-74aa overexpression promoted G1-phase to S-phase progression (Fig. 4C). CCK-8 and EdU staining also revealed that the proliferation of chicken myoblasts was significantly promoted upon MPD-74aa overexpression (Fig. 4D and E). Immunofluorescence staining revealed that MPD-74aa overexpression significantly inhibited myoblast differentiation, as indicated by suppressed fusion with multinucleated myotubes (Fig. 4F). Accordingly, compared with those in the control group, the expression of marker genes closely associated with myoblast differentiation (*MYOD*, *MYOG*, and *MYHC*) was markedly downregulated in MPD-74aa-overexpressing chicken myoblasts (Fig. 4G). Moreover, MPD-74aa overexpression suppressed the expression of the MYHC protein and promoted the expression of the CDK1 protein (Fig. 4H and I). Taken together, these results suggest that MPD-74aa can promote the proliferation and inhibit the differentiation of myoblasts in chickens.

### MPD-74aa interacts with the CDK1 protein

**Fig. 5:**
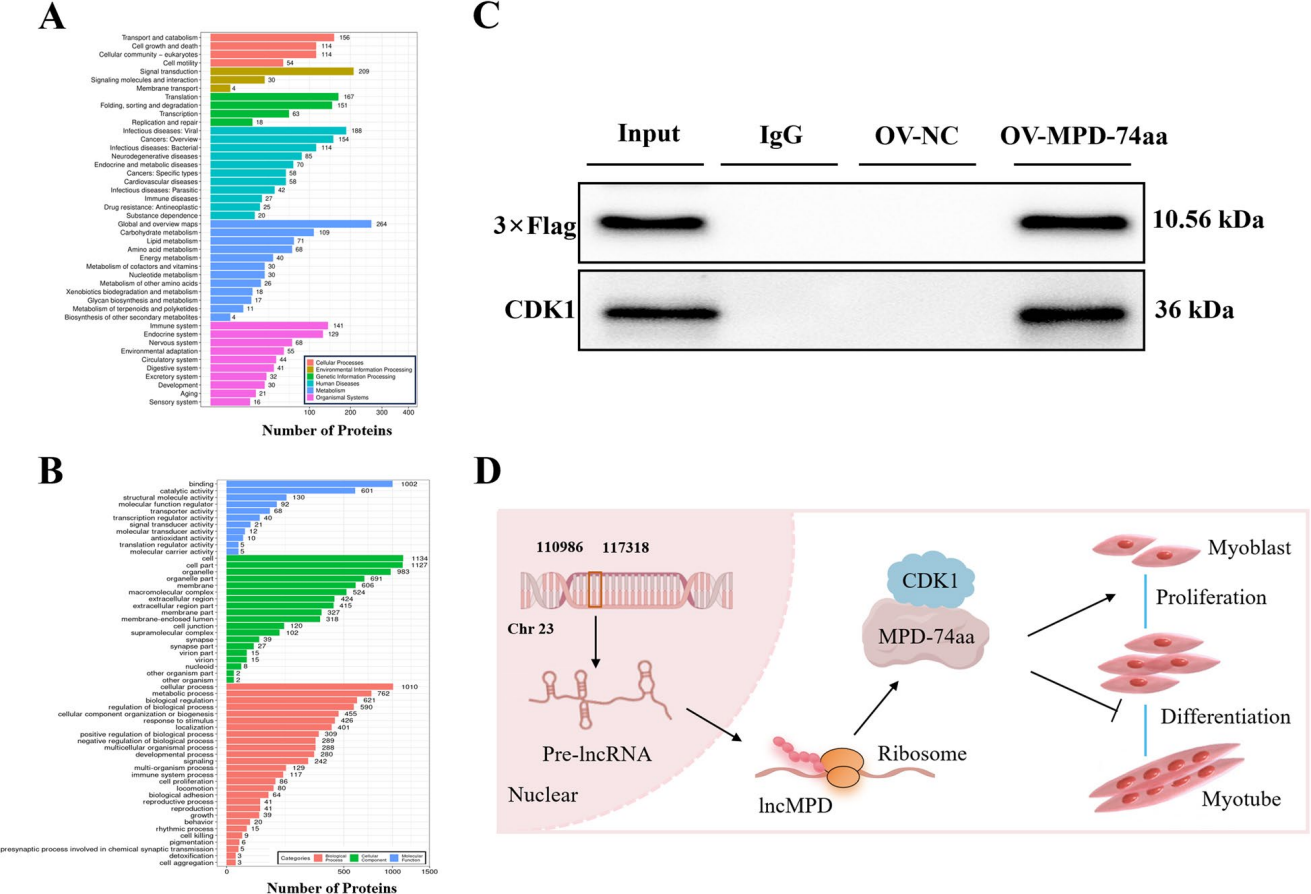
MPD-74aa interacts with the CDK1 protein. **A** KEGG enrichment plot for interacting proteins. **B** GO enrichment plot for interacting proteins. **C** Detection of pcDNA3.1–3 × Flag and CDK1 protein expression in the pull-down complex of MPD-74aa. **D** Schematic representation of the regulatory mechanism mediated by MPD-74aa encoded by lncMPD in chicken myogenesis

To investigate the specific mechanism by which MPD-74aa exerts its function, we overexpressed MPD-74aa in myoblasts and subsequently employed Co-IP to identify binding proteins. The results revealed that 1,540 proteins interacted with MPD-74aa. KEGG enrichment analysis of these interacting proteins revealed their predominant enrichment in signaling pathways related to cell proliferation, apoptosis, cell motility, and translation (Fig. 5A, B and Tables S5–S7). To further identify the key functional proteins that directly interact with the target protein MPD-74aa, we conducted targeted WB validation experiments based on coimmunoprecipitation combined with mass spectrometry analysis. The results confirmed that MPD-74aa can specifically bind to the core cell cycle regulatory protein CDK1. These findings suggest that MPD-74aa may positively regulate cell proliferation by directly binding to CDK1 (Fig. 5C). In summary, lncMPD encodes a 10.56 kDa peptide (MPD-74aa) that promotes myoblast proliferation and inhibits differentiation by directly interacting with CDK1 and enhancing its expression (Fig. 5D).

## Discussion

Research has shown that some lncRNAs can encode small peptides with important biological functions, which have become a research hotspot in recent years [53, 54]. Multiple findings have demonstrated novel lncRNA-encoded peptides, their important biological significance in cancer, and their novel regulatory mechanisms. For instance, the RBRP peptide upregulated in metastatic colorectal cancer can bind to the IGF2BP1-c-Myc mRNA complex and stabilize c-Myc mRNA, thereby promoting tumor progression [55]. The FORCP peptide inhibits cell proliferation and induces apoptosis in response to endoplasmic reticulum stress [56]. Moreover, the functional specificity of lncRNAs is closely linked to their temporal expression patterns. Analyzing expression profiles across different developmental stages of myoblasts serves as an effective strategy for identifying functionally significant lncRNAs [51, 57]. In the research field of skeletal muscle development regulation, an increasing number of lncRNA-encoded peptides with biological functions have been reported. The lncRNA-encoded DWORF peptide in mice participates in muscle function regulation by enhancing SERCA activity [34], whereas the SPAR peptide modulates the muscle regeneration process by negatively regulating the mTORC1 signaling pathway [58]. Furthermore, lncMGPF is highly conserved across species, including humans, mice and pigs, and its encoded functional peptide targets myogenic differentiation pathways, effectively promoting myoblast differentiation, increasing muscle mass and accelerating muscle injury repair [59]. Collectively, these findings suggest that lncRNA-encoded peptides play a nonnegligible regulatory role in skeletal muscle development.

In this study, a multi-omics strategy combining RNA-seq and Ribo-seq was employed to systematically screen the candidates in chicken myoblasts at different developmental time points. By integrating the 178 DE-lncRNAs identified by RNA-seq with the 18 translational capable lncRNAs validated by Ribo-seq, we obtained three key lncRNAs exhibiting both differential expression and translational potential. Among three candidates, lncMPD was highly expressed in proliferating myoblasts and encodes a 74-aa peptide, designated MPD-74aa. Expression profiling revealed that MPD-74aa exhibited high expression levels in proliferating myoblasts and was highly expressed in E10 and E12 embryonic stages, especially in the leg and pectoralis muscle tissues at E12. This expression pattern strongly suggests its potential involvement in regulating skeletal muscle embryonic development and myoblast differentiation. Mass spectrometry and WB experiments further confirmed that MPD-74aa encodes a peptide with a molecular weight of 10.56 kDa. Gain-of-function experiments revealed that this peptide significantly promotes myoblast proliferation while inhibiting differentiation, revealing its dual regulatory role in myogenesis. At the mechanistic level, co-IP experiments revealed 1,540 proteins that interact with MPD-74aa (primarily enriched in cell proliferation and apoptosis-related signaling pathways). Subsequent WB validation further confirmed its direct binding to CDK1, a key regulator of the G2/M phase transition to promote cell proliferation, which also inhibits myoblast differentiation by phosphorylating MyoD at Ser200 to reduce its stability and transcriptional activity [1, 60, 61]. On this basis, we speculate that MPD-74aa may forms a complex with CDK1 via its encoded peptide, thereby maintaining CDK1 protein stability to promote myoblast proliferation and inhibit myoblast differentiation. This discovery establishes the first molecular link between lncRNA-encoded peptides and cell cycle regulation during myogenesis, providing a novel perspective for research in this field. However, the specific molecular mechanism by which this lncRNA and its encoded small peptide MPD-74aa exert their functions still needs to be elucidated.

## Conclusion

In summary, through the integration of RNA-seq and Ribo-seq analyses, this study identified three DE-lncRNAs with translational potential. Among them, lncMPD was confirmed to encode a 10.56 kDa peptide (MPD-74aa). This peptide can promote the proliferation of myoblasts and inhibit their differentiation. The mechanism involves its direct interaction with CDK1 and promotion of CDK1 expression to regulate cellular processes. This study preliminarily elucidated the biological characteristics and molecular mechanism of the polypeptide encoded by this lncRNA at the functional level, providing a new perspective for functional research on lncRNAs and broadening our understanding of the regulatory mechanism of CDK1. These findings provide novel targets and a theoretical basis for research on skeletal muscle development and poultry breeding.

## Supplementary Information

Additional file 1: Fig. S1 Screening of skeletal muscle development-related lncRNAs by RNA-seq. (A) De-lncRNAs heatmap. (B) De-lncRNAs target gene GO functional enrichment. (C) De-lncRNAs target gene KEGG functional enrichment. (D) WGCNA module partitioning for all lncRNAs.

Additional file 2: Fig. S2 Screening coding-potential lncRNAs by RNA-seq and Ribo-seq integration. (A) Heatmap of sample correlation for lncRNA transcriptome sequencing. (B) Intergroup difference gene volcano plot: the blue regions indicate downregulated genes, and the red regions indicate upregulated genes. (C) Score distribution of mORFs and sORFs. The green plot indicates lncORFs, the red plot indicates uORFs, the blue plot indicates dORFs, and the purple plot indicates mORFs.

Additional file 3: Table S1. Primer list.

Additional file 4: Table S2. 444 common lncRNAs were obtained by different stages.

Additional file 5: Table S3. 178 DE-lncRNAs were included in three analyses.

Additional file 6: Table S4. 3545 differentially translated genes were obtained by D1 vs. D2.

Additional file 7: Table S5. 1540 interacting protein.

Additional file 8: Table S6. Enrichment analysis of 1540 interacting protein KEGG.

Additional file 9: Table S7. Enrichment analysis of 1540 interacting protein GO.

## Data Availability

All raw transcriptome data has been deposited in SRA (NCBI PRJNA909444 and PRJNA1390989). The data that support the findings of this study are available from the corresponding author upon reasonable request.

## Abbreviations

AA: Arbor Acres
CCK-8: Cell Counting Kit-8
Co-IP: Co-Immunoprecipitation
CDK1: Cyclin-dependent kinase 1
DMEM: Dulbecco’s Modified Eagle Medium
DE-lncRNAs: Differentially expressed lncRNAs
EdU: 5-Ethynyl-2-deoxyuridine
LncRNAs: Long noncoding RNAs
qRT-PCR: Quantitative real-time PCR
RRS: Ribosome release score
Ribo-seq: Ribosome sequencing
SDS-PAGE: Sodium dodecyl sulfate-polyacrylamide gel electrophoresis
WB: Western blot
WGCNA: Weighted gene co-expression network analysis
