# Supplementary figures and images for: Integrative analysis of RNA-seq and Ribo-seq reveals that lncRNA regulates chicken myogenesis through encoding peptide
Source: J Anim Sci Biotechnol. 2026 May 29;17:103. doi: 10.1186/s40104-026-01421-y (PMC13220464; doi:10.1186/s40104-026-01421-y)

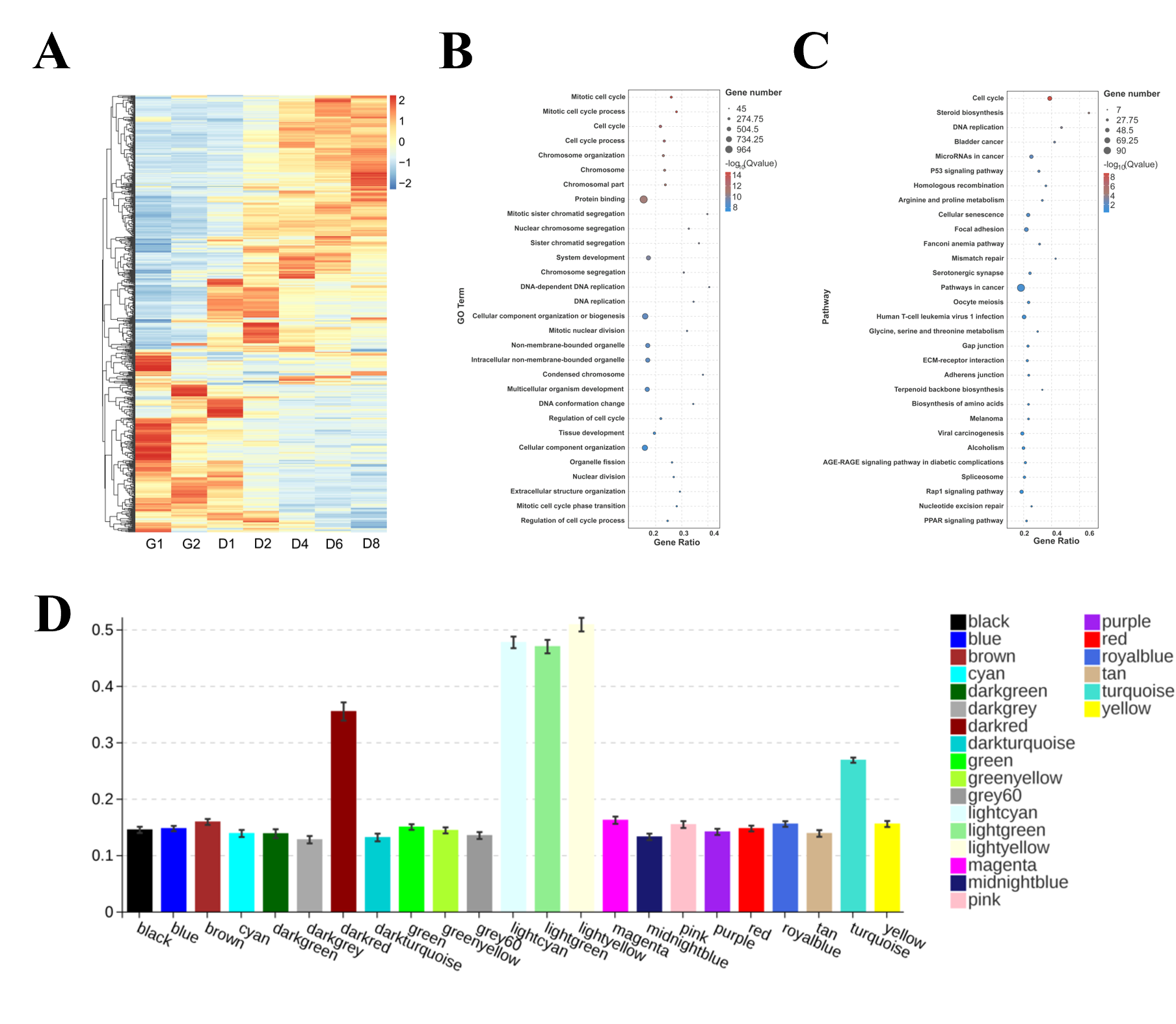

Supplement: Supplementary file 1 — Additional file 1: Fig. S1 Screening of skeletal muscle development-related lncRNAs by RNA-seq. (A) De-lncRNAs heatmap. (B) De-lncRNAs target gene GO functional enrichment. (C) De-lncRNAs target gene KEGG functional enrichment. (D) WGCNA module partitioning for all lncRNAs. [file 40104_2026_1421_MOESM1_ESM.tif]

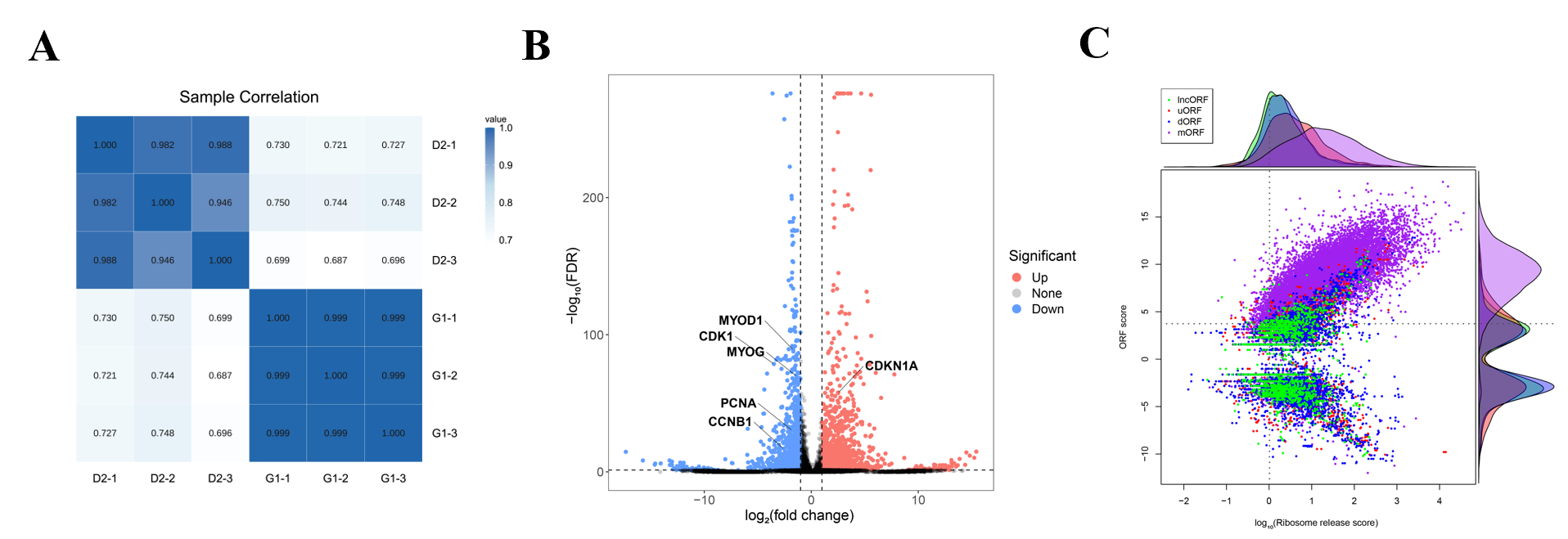

Supplement: Supplementary file 2 — Additional file 2: Fig. S2 Screening coding-potential lncRNAs by RNA-seq and Ribo-seq integration. (A) Heatmap of sample correlation for lncRNA transcriptome sequencing. (B) Intergroup difference gene volcano plot: the blue regions indicate downregulated genes, and the red regions indicate upregulated genes. (C) Score distribution of mORFs and sORFs. The green plot indicates lncORFs, the red plot indicates uORFs, the blue plot indicates dORFs, and the purple plot indicates mORFs. [file 40104_2026_1421_MOESM2_ESM.tif]
