# Supplementary material for: Integrative analysis of RNA-seq and Ribo-seq reveals that lncRNA regulates chicken myogenesis through encoding peptide
Source: J Anim Sci Biotechnol. 2026 May 29;17:103. doi: 10.1186/s40104-026-01421-y (PMC13220464; doi:10.1186/s40104-026-01421-y)
Supplement: Supplementary file 3 — Additional file 3: Table S1. Primer list. [file 40104_2026_1421_MOESM3_ESM.docx]

**Table S1 Primer list**

| **Target** | **Primer** | **Sequence（5´→3´）** | **TM,** °**C** |
| --- | --- | --- | --- |
| MPD-74aa | F | GACGAATCCACCGTTGGGAC | 60 |
|  | R | AAATCATTCTGCCCTTGCTCCTG |  |
| *ENSGALT00000100638* | F | AGAGCACAGGTGGATTTGCC | 60 |
|  | R | GGTCCCCGCTACAACGATTT |  |
| *GAPDH* (qRT-PCR) | F | GAACATCATCCCAGCGTCCA | 60 |
|  | R | CGGCAGGTCAGGTCAACAAC |  |
| *p21* (qRT-PCR) | F | GAAGAGTTGTCCACGATAAGC | 60 |
|  | R | TTCCAGTCCTCCTCAGTCC |  |
| *CCND1* (qRT-PCR) | F | CAGAAGTGCGAAGAGGAAGT | 60 |
|  | R | CTGATGGAGTTGTCGGTGTA |  |
| *CDK1* (qRT-PCR) | F | TAATAGATGACAAAGGGGT | 60 |
|  | R | GAGTGGAATACAGAGCAGA |  |
| *PCNA* (qRT-PCR) | F | AGCACCAAATCAGGAAAAG | 60 |
|  | R | GCACAGGAGATGACAACAG |  |
| *MYOD* (qRT-PCR) | F | GCTACTACACGGAATCACCAAAT | 56 |
|  | R | CTGGGCTCCACTGTCACTCA |  |
| *MYOG* (qRT-PCR) | F | CGGAGGCTGAAGAAGGTGAA | 56 |
|  | R | CGGTCCTCTGCCTGGTCAT |  |
| *MYHC* (qRT-PCR) | F | CTCCTCACGCTTTGGTAA | 56 |
|  | R | TGATAGTCGTATGGGTTGGT |  |

TM refers to the annealing temperature; F and R refer to the forward and reverse primers, respectively
